# DJ-1 protects retinal pericytes against high glucose-induced oxidative stress through the Nrf2 signaling pathway

**DOI:** 10.1038/s41598-020-59408-2

**Published:** 2020-02-12

**Authors:** Wanpeng Wang, Han Zhao, Baihua Chen

**Affiliations:** 1 Department of Ophthalmology, the second Xiangya Hospital of Central South University, Changsha, Hunan 410011 China; 2 Hunan Clinical Research Center of Ophthalmic Disease, Changsha, Hunan 410011 China; 3 Department of Ophthalmology, Xiangya Hospital, Central South University, Changsha, Hunan 410008 China

**Keywords:** Apoptosis, RNAi

## Abstract

Oxidative stress has been associated with the etipathogenesis of Diabetic retinopathy (DR). Studies have shown that DJ-1 plays an important role in regulating the reactive oxygen species (ROS) production and resistance to oxidative stress-induced apoptosis. This study aimed to investigate whether DJ-1 upregulates oxidative stress and prevents damage to retinal capillary pericytes by increasing antioxidant capacity through the Nuclear factor erythroid 2-related factor 2 (Nrf2) signaling pathway. Nrf2 is a redox-sensitive transcription factor that encode antioxidant enzymes and phase II metabolic enzymes, activation of Nrf2 functions is one of the critical defensive mechanisms against oxidative stress in many tissues. Our results showed after DJ-1 overexpression, apoptosis of rat retinal pericytes (RRPs) decreased, the ratio of B-cell lymphoma-2 (Bcl-2) to BCL2-Associated X Protein (BAX) increased, the production of ROS decreased, and the protein expression and activity of manganese superoxide dismutase (MnSOD, also called SOD2) and catalase (CAT) increased. DJ-1 overexpression activated Nrf2 expression, however, after Nrf2 silencing, apoptosis of RRPs increased, the ratio of Bcl-2 to BAX decreased, the production of ROS increased, the protein expression of MnSOD and CAT decreased, and the expression of heme oxygenase-1 (HO-1), NADP(H) quinone oxidoreductase (NQO1), glutamate-cysteine ligase catalytic subunit (GCLC) and modifier subunit (GCLM) decreased. These data suggest that enhancement of the Nrf2 pathway is a potential protective strategy for the treatment of DR. Therefore, DJ-1 may prevent high glucose-induced oxidative stress and RRPs apoptosis through the Nrf2 signaling pathway, thereby preventing the early onset and progression of DR.

## Introduction

Diabetic retinopathy (DR) is the most common microvascular disease associated with diabetic complications. It has been reported that the global incidence of diabetes is expected to rise sharply to 592 million by 2035^1^. At present, the number of patients with diabetes has reached 90 million in China^2^. After 20 years of diabetes, nearly all patients with type I diabetes, 80% of patients with type 2 diabetes requiring insulin treatment and 50% of those not requiring insulin treatment exhibit some degree of DR^3^.

Long-term hyperglycemia can damage the blood-retinal barrier in the early stages of DR. The major biochemical changes induced by hyperglycemia, which are implicated in the pathogenesis of DR, include increased polyol pathway flux, increased intracellular advanced glycation end product (AGE) formation, protein kinase C (PKC) activation, hexosamine pathway activation, poly(adenosine diphosphate ribose) polymerase (PARP) activation, increased oxidative stress and reactive oxygen species (ROS) production, and nuclear factor kappa-light-chain-enhancer of activated B cell (NF-κB) activation^4–7^. Some studies have suggested that high glucose-induced ROS overproduction may be the basis of the involvement of these classical pathways, as oxidative stress plays a very important role in the development of DR^8,9^. Under high-glucose stimulation, ROS production increases, while superoxide dismutase (SOD), catalase (CAT) and other antioxidant activity decreases.Meanwhile, cell membrane integrity is disrupted, apoptosis increases, and microvascular damage and barrier function are destroyed. As key factors in DR initiation, ROS have become important targets for the prevention and treatment of DR.

The DJ-1 (PARK7) gene encodes a highly conserved 189-amino acid protein with a molecular weight of 20 kD that has protease and chaperone functions^10,11^. DJ-1 can be expressed in peripheral tissues, neurons and glial cells. Studies have shown that the DJ-1 protein can be transported to the mitochondrial outer membrane and thus prevent oxidative stress damage in the body. The functions of DJ-1 in cancer and Parkinson’s disease have been intensively studied^12–14^. Although the function of DJ-1 is still not fully understood, it has been found that DJ-1 plays an important role in regulating the ROS production and resistance to oxidative stress-induced apoptosis^15^. With regards to ocular diseases, studies have shown that DJ-1 plays a role in anti-oxidative stress mechanisms in Fuchs corneal endothelial dystrophy and age-related macular degeneration^10,16^ and that DJ-1-deficient retina changes are associated with increased oxidative stress. However, whether and how DJ-1 regulates oxidation and plays a protective role in DR remain unknown.

Nuclear factor erythroid 2-related factor 2 (Nrf2) is a redox-sensitive transcription factor that binds to antioxidant response elements (AREs) located in the promoter regions of many genes that encode antioxidant enzymes and phase II metabolic enzymes^17^. Initial evidence demonstrating the role of Nrf2 in induction of antioxidant/detoxifying enzymes came from studies on the participation of Nrf2 in ARE-mediated expression of a variety of antioxidant genes, including heme oxygenase-1 (HO-1), NADP(H) quinone oxidoreductase (NQO1), and glutamate-cysteine ligase catalytic subunit (GCLC) and modifier subunit (GCLM)^18^. Activation of Nrf2 functions is one of the critical defensive mechanisms against oxidative stress in many tissues. Another study indicated that Nrf2 participates in an important protective mechanism regulating the progression of DR and suggested that enhancement of the Nrf2 pathway is a potential protective strategy for the retina^19^. Therefore, we explored whether DJ-1 upregulates oxidative stress and prevents damage to retinal capillary pericytes by increasing antioxidant capacity through the Nrf2 signaling pathway.

## Results

### High glucose induced rat retina pericytes (RRPs) apoptosis, upregulated apoptotic gene expression, and increased oxidative stress

**Figure 1:**
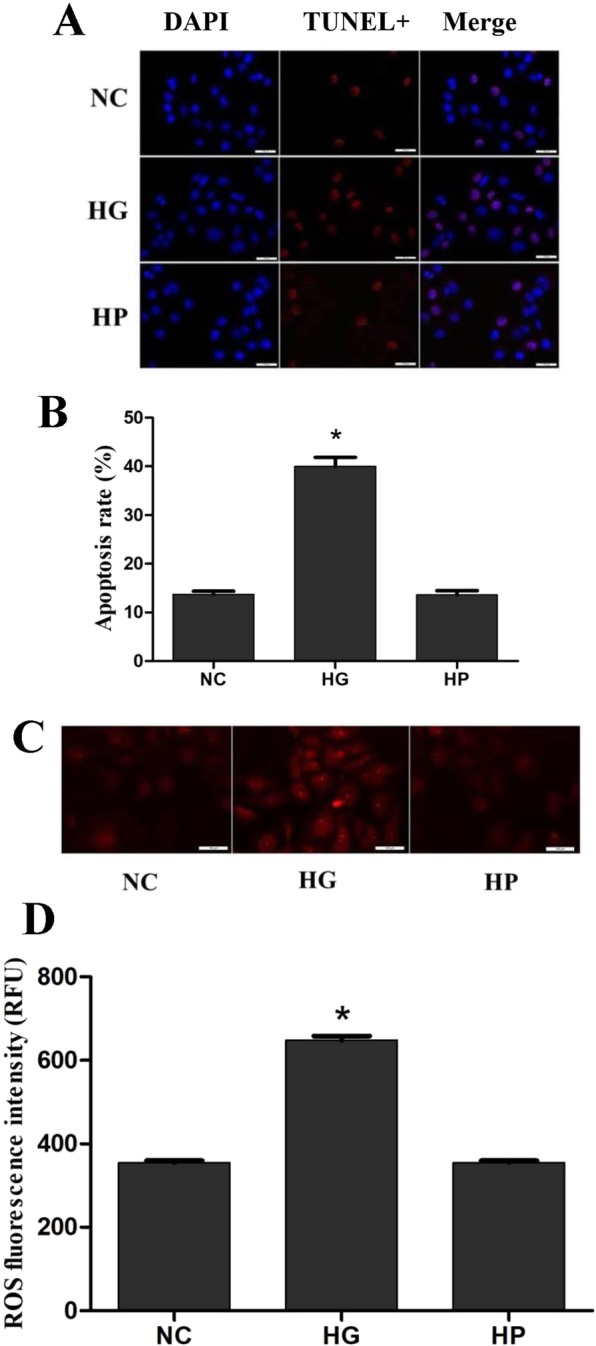
High glucose induces RRPs apoptosis and oxidative stress. RRPs were cultured for 2 days in different media. (**A**) High glucose induces RRPs apoptosis detected using TUNEL staining. Blue indicates DAPI-stained nuclei, while red indicates TUNEL-positive cells. (**B**) Quantification of TUNEL staining showing that more RRPs apoptosis in the HG group than the NC and HP groups. (**C**) Intracellular ROS levels were measured with the fluorescent probe DCFH-DA, and images were obtained by fluorescence microscopy. (**D**) The ROS fluorescence intensity values were calculated using Adobe Photoshop version 7.0. The ROS levels of RRPs in the HG group were significantly higher than those of RRPs in the NC group. RFU: relative fluorescence unit. All data are presented as means ± SEM(n = 3/group). ^*^P < 0.05 vs NC, HP.

To identify any increases in apoptosis induced by high glucose, we first measured the levels of apoptosis using a TdT-mediated dUTP Nick-End Labeling (TUNEL) staining assay (Fig. 1A,B). After 2 days of stimulation of RRPs in a high-glucose (30 mM) environment, the apoptosis percentage of RRPs in the high glucose (HG) group was 41.4%, while that in the normal control (NC) group was 14.3%. There was no significant difference in RRPs apoptosis between the hypertonic (HP) group and the NC group, indicating that osmotic pressure had no effect on this parameter.

To determine whether high glucose-induced apoptosis was associated with corresponding changes in ROS production, we used a ROS assay to examine the levels of ROS. We found that the ROS levels in RRPs in the HG group were significantly increased (Fig. 1C,D), suggesting that high glucose strongly induced ROS accumulation, while there was no significant difference between the HP group and the NC group, indicating that osmotic pressure had no effect on intracellular ROS levels.

**Figure 2:**
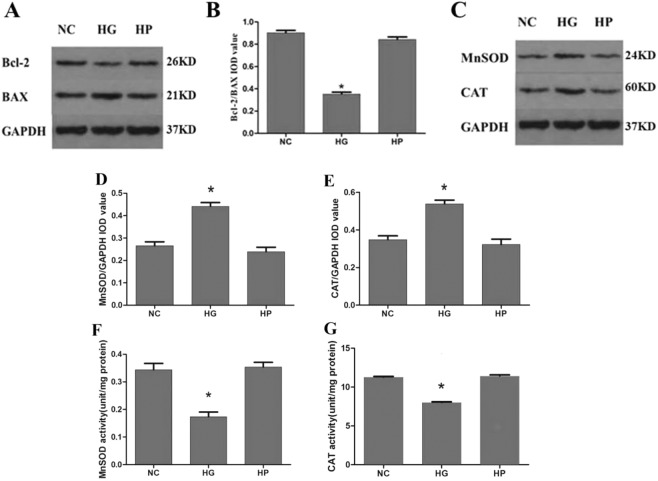
The expression levels of Bcl-2, BAX, MnSOD and CAT and the activity of MnSOD and CAT in RRPs exposed to high-glucose conditions. (**A**,**B**) High glucose significantly increased BAX levels and reduced Bcl-2 levels. The Bcl-2/BAX ratio therefore decreased. (**C**–**E**) The expression levels of MnSOD and CAT were higher in RRPs treated with high glucose than in NC and HP group RRPs. (**F**,**G**) The activity of MnSOD and CAT was decreased under high-glucose conditions. All data are presented as means ± SEM(n = 3/group). ^*^P < 0.05 vs NC, HP.

Apoptosis can be regulated by antiapoptotic genes and apoptotic genes. The ratio of the antiapoptotic protein Bcl-2 to the apoptotic protein BAX was determined to assess the survival and apoptosis of cells stimulated by apoptotic factors. We performed western blot analysis to examine the expression levels of Bcl-2, BAX, MnSOD and CAT in RRPs exposed to normal conditions, high-glucose conditions or hypertonic conditions. High glucose treatment for 2 days significantly upregulated BAX levels and downregulated Bcl-2 levels, and the Bcl-2/BAX ratio was decreased to approximately 0.4 times the control value (Fig. 2A,B). The expression levels of MnSOD and CAT were higher in RRPs treated with high glucose than in NC group RRPs (Fig. 2C–E). However, the activity of MnSOD and CAT was decreased under high-glucose conditions (Fig. 2F,G). Together, these results suggest that apoptosis and oxidative stress were increased in RRPs under high-glucose conditions.

### Overexpression of DJ-1 enhanced DJ-1 expression in RRPs, upregulated antiapoptotic gene expression, and reduced RRPs apoptosis

**Figure 3:**
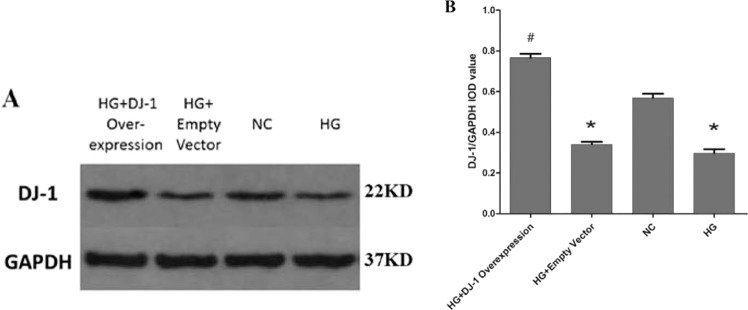
Western blot analysis of DJ-1 protein expression after DJ-1 overexpression. (**A**,**B**) DJ-1 protein expression in the DJ-1 overexpression group was significantly higher than that in the other groups, indicating that we could transfect RRPs with the pcDNA3.1-myc-DJ-1 plasmid to successfully overexpress DJ-1. All data are presented as means ± SEM(n = 3/group). ^*^P < 0.05 vs NC; ^#^P < 0.05 vs NC, HG.

To determine whether DJ-1 plays an important role in the apoptosis of RRPs, we transfected RRPs with the pcDNA3.1-myc-DJ-1 plasmid to overexpress DJ-1. Then, we performed western blot analysis to examine the expression levels of DJ-1; as expected, the results indicated that this method successfully induced DJ-1 overexpression (Fig. 3A,B).

**Figure 4:**
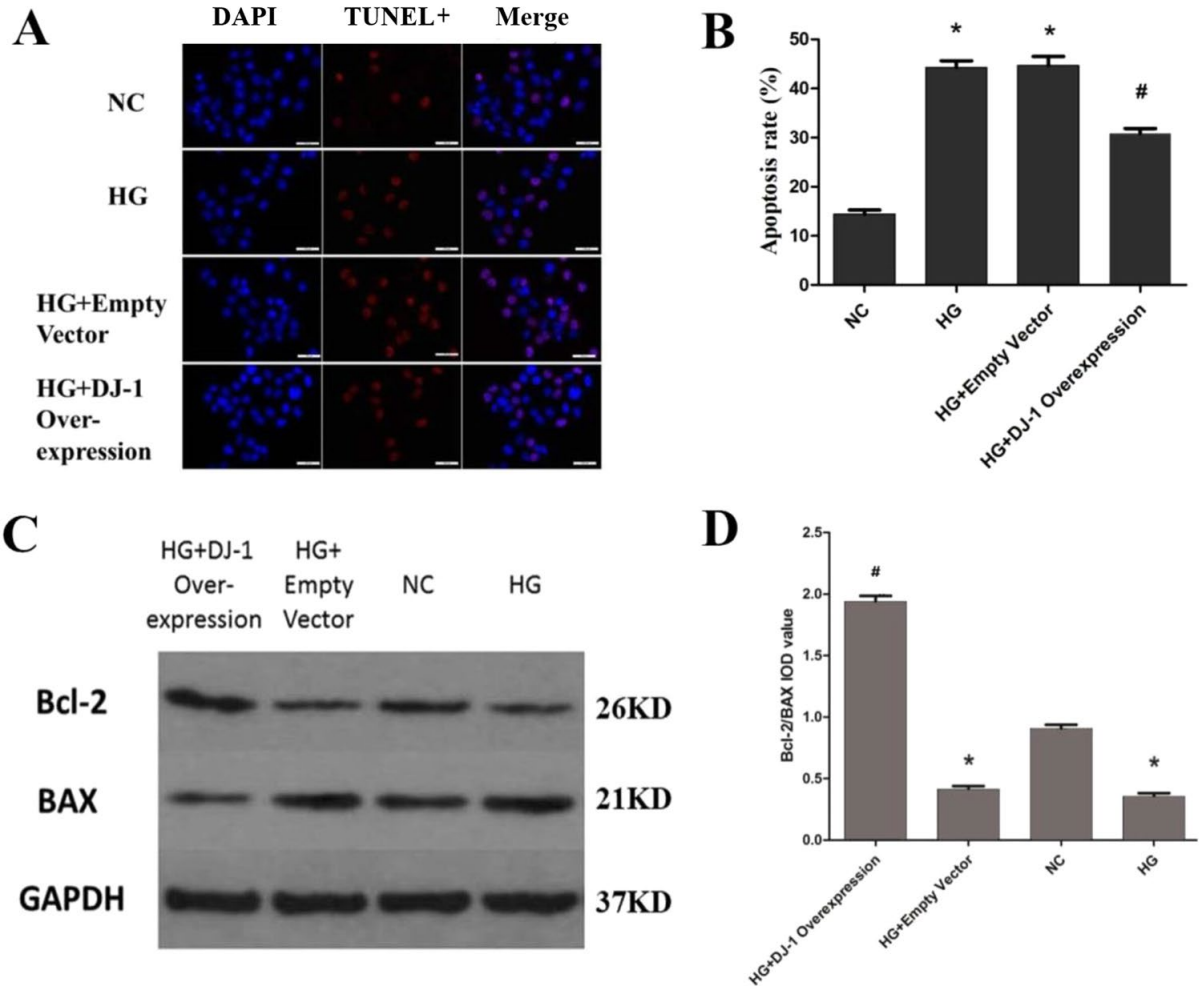
DJ-1 reduces RRPs apoptosis. (**A**) DJ-1 overexpression reduces RRPs apoptosis. Blue indicates DAPI-stained nuclei, while red indicates TUNEL-positive cells. (**B**) Quantification of TUNEL staining showing that the DJ-1 overexpression group showed lower apoptosis levels than the HG group. (**C**,**D**) After DJ-1 overexpression, Bcl-2 protein levels increased, BAX protein levels decreased, and the Bcl-2/BAX ratio increased. All data are presented as means ± SEM(n = 3/group). ^*^P < 0.05 vs NC; ^#^P < 0.05 vs NC, HG.

Next, to determine whether DJ-1 overexpression reduces apoptosis, we measured the level of apoptosis by TUNEL staining and examined the expression levels of Bcl-2 and BAX in RRPs under different conditions. The results demonstrated that in DJ-1-overexpressing RRPs, apoptosis was decreased by 0.7-fold under high-glucose conditions (Fig. 4A,B). We also found that DJ-1 overexpression upregulated Bcl-2 protein levels and downregulated BAX protein levels (Fig. 4C,D), demonstrating that DJ-1 plays a protective role against apoptosis in RRPs stimulated by high glucose.

### DJ-1 prevented high glucose-induced ROS production in RRPs, enhanced antioxidant enzyme expression and activity, and unregulated Nrf2 and downstream antioxidant gene expression

**Figure 5:**
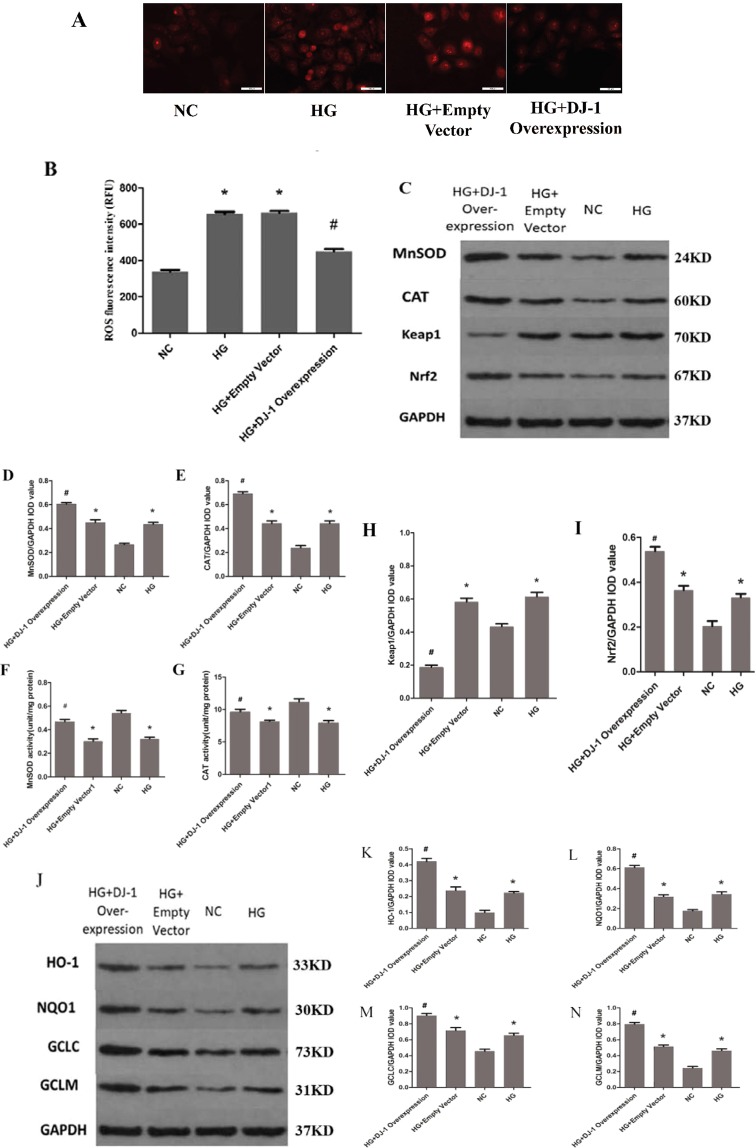
DJ-1 can prevent oxidative stress in RRPs under high glucose. (**A**,**B**) Intracellular ROS levels were measured with the fluorescent probe DCFH-DA, and images were obtained by fluorescence microscopy. The ROS fluorescence intensity values were calculated using Adobe Photoshop version 7.0. DJ-1 overexpression reduced ROS production in RRPs under high-glucose conditions. (**C**–**E**) After DJ-1 overexpression, the expression levels of MnSOD and CAT were increased in RRPs exposed to high glucose. (**F**,**G**) After DJ-1 overexpression, the activity of MnSOD and CAT was increased in RRPs exposed to high glucose. (**H**,**I**) DJ-1 activated Nrf2 and reduced Keap1 expression. (**J**–**N**) DJ-1 enhanced the protein expression levels of HO-1, NQO1, GCLC and GCLM in RRPs. All data are presented as means ± SEM(n = 3/group). ^*^P < 0.05 vs NC; ^#^P < 0.05 vs NC, HG.

We next determined whether DJ-1 reduces apoptosis by protecting against oxidative stress. After DJ-1 overexpression, ROS production was decreased by 0.66-fold in RRPs under high-glucose conditions (Fig. 5A,B). These results indicate that DJ-1 can enhance the expression levels and activity of MnSOD and CAT (Fig. 5C–G), suggesting that DJ-1 can protect against oxidative stress in RRPs to decrease apoptosis. However, how DJ-1 modulates oxidative stress remains unclear and needs further study.

Nrf2 is a major transcription factor associated with oxidative stress and can modify the basal and inducible expression of several antioxidant genes. Kelch-like ECH-associated protein 1 (Keap1) is a major Nrf2 repressor that interacts with Nrf2 and mediates its ubiquitination and proteolysis. To determine whether DJ-1 can regulate the Nrf2 signaling pathway, we performed western blot analysis to examine the expression levels of Keap1 and Nrf2 after DJ-1 overexpression. The results indicated that DJ-1 can activate Nrf2 and reduce Keap1 expression (Fig. 5C,H,I).

Then, we investigated the mechanism by which DJ-1 could regulate oxidative stress. We hypothesized that DJ-1 could activate Nrf2-targeted antioxidant genes; therefore, we performed western blot analysis to examine the expression levels of such genes, including HO-1, NQO1, GCLC and GCLM, under different conditions. The protein levels of HO-1, NQO1, GCLC, and GCLM were significantly higher in the DJ-1 overexpression group than in the other groups (Fig. 5J–N), which indicated that DJ-1 can activate the expression of Nrf2-targeted antioxidant genes.

### After inhibition of Nrf2, the protective effect of DJ-1 against RRPs apoptosis and oxidative stress was weakened

**Figure 6:**
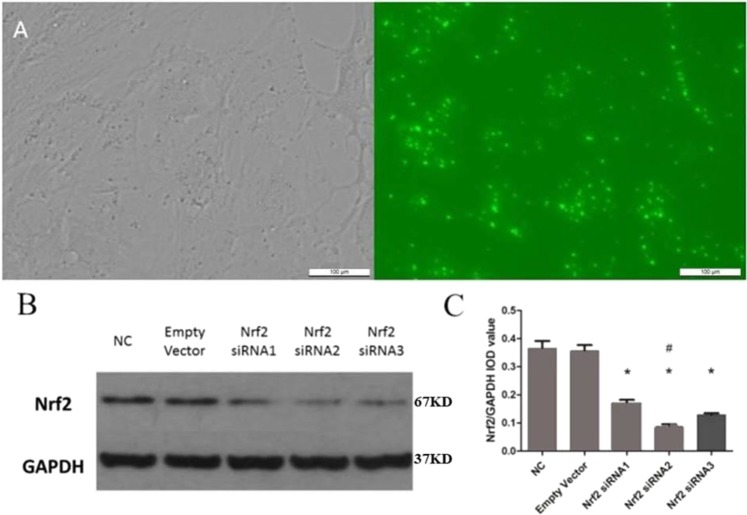
Nrf2 siRNA was transfected into RRPs and successfully reduced Nrf2 protein expression. (**A**) RRPs transfected with fluorescent empty vector siRNA revealed significant FAM fluorescence after 6 hours, indicating that Nrf2 siRNA could also be successfully transfected into RRPs. (**B**,**C**) Compared with the NC and empty vector groups, the Nrf2 siRNA-transfected group demonstrated reduced protein expression of Nrf2, and Nrf2 siRNA2 provided the highest silencing efficiency. All data are presented as means ± SEM(n = 3/group). ^*^P < 0.05 vs NC; ^#^P < 0.01 vs NC.

**Figure 7:**
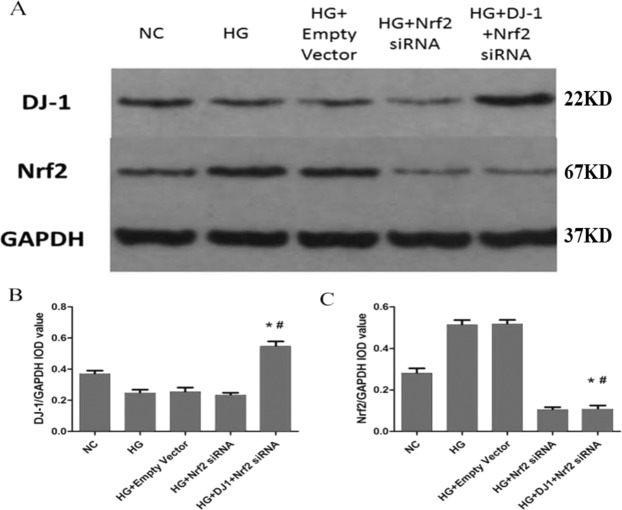
The expression levels of DJ-1 and Nrf2 after DJ-1 overexpression and Nrf2 silencing. (**A**,**B**) The expression of DJ-1 in RRPs was higher in the DJ-1 overexpressing and Nrf2-silenced group than in the Nrf2-silenced group, which indicated that Nrf2 silencing had no effect on DJ-1 protein expression. (**A**,**C**) Nrf2 expression in RRPs was significantly lower in the Nrf2-silenced group and in the DJ-1-overexpressing and Nrf2-silenced group than in the other groups. All data are presented as means ± SEM(n = 3/group). ^*^P < 0.05 vs NC; ^#^P < 0.05 vs HG.

DJ-1 was found to confer protection and increase cell survival under high-glucose conditions. To investigate whether the Nrf2 signaling pathway is involved in the cellular protection mediated by DJ-1 and to determine whether Nrf2 exerts beneficial effects in RRPs against apoptosis and oxidative stress, we used siRNAs to modulate Nrf2 expression (Fig. 6A–C). We first confirmed that Nrf2 siRNAs could effectively reduce the expression of Nrf2 and selected the most efficient siRNA sequence for use in subsequent experiments. We then evaluated the protein expression levels of DJ-1 and Nrf2 after DJ-1 overexpression combined with Nrf2 silencing (Fig. 7A–C).

**Figure 8:**
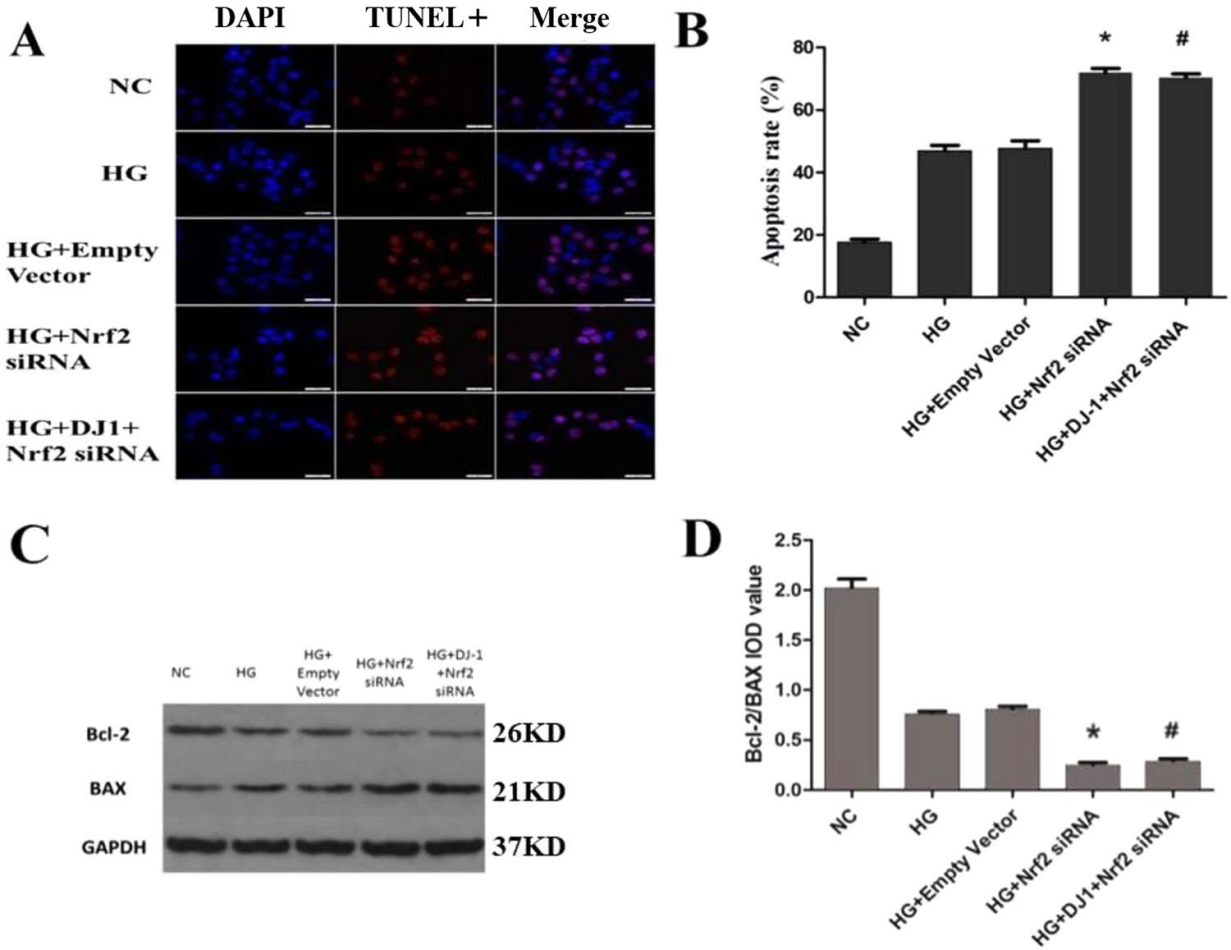
Increased apoptosis of RRPs after Nrf2 silencing. (**A**) Nrf2 silencing increase apoptosis of RRPs. Blue indicates DAPI-stained nuclei, while red indicates TUNEL-positive cells. (**B**) Quantification of TUNEL staining showing that RRPs apoptosis was increased after Nrf2 silencing. (**C**,**D**) After Nrf2 silencing, Bcl-2 expression was significantly decreased, BAX expression was significantly increased, and the Bcl-2/BAX ratio was decreased. All data are presented as means ± SEM(n = 3/group). ^*^P < 0.05 vs NC; ^#^P < 0.05 vs HG.

**Figure 9:**
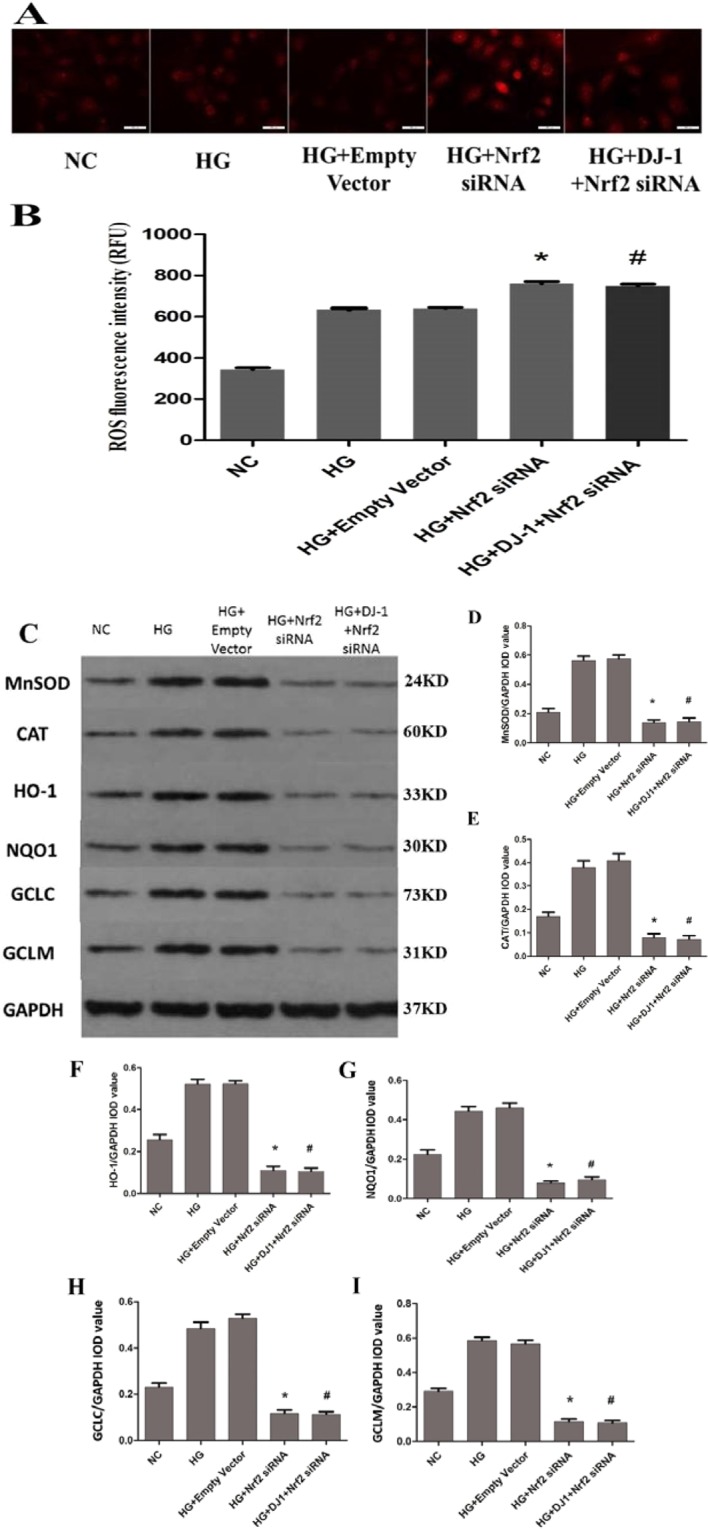
Increased oxidative stress in RRPs after Nrf2 silencing. (**A**,**B**) Intracellular ROS levels were measured with the fluorescent probe DCFH-DA, and images were obtained by fluorescence microscopy. The ROS fluorescence intensity values were calculated using Adobe Photoshop version 7.0. After Nrf2 silencing, more ROS were produced in the Nrf2-silenced group than in the HG group. (**C**) The protein expression levels of MnSOD, CAT, HO-1, NQO1, GCLC and GCLM in each group of RRPs after Nrf2 silencing. (**D**,**E**) After Nrf2 silencing, the expression levels of MnSOD and CAT were decreased in RRPs exposed to high glucose. (**F**–**I**) Nrf2 silencing reduced the protein levels of HO-1, NQO1, GCLC and GCLM in RRPs, and DJ-1 overexpression had no effect on the expression of HO-1, NQO1, GCLC and GCLM after Nrf2 silencing. All data are presented as means ± SEM(n = 3/group). ^*^P < 0.05 vs NC; ^#^P < 0.05 vs HG.

The apoptosis percentage was higher in the Nrf2-silenced group (72.2%) than in the HG group (50.0%; Fig. 8A,B). Western blot analysis confirmed the increase in BAX and the decrease in Bcl-2 protein levels in Nrf2 siRNA-transfected cells (Fig. 8C,D). Similarly, ROS production was significantly higher in RRPs with Nrf2 gene knockdown than in HG group RRPs (Fig. 9A,B). In contrast, the protein expression levels of MnSOD and CAT were decreased after Nrf2 silencing (Fig. 9C–E). After Nrf2 gene silencing, we further analyzed the expression levels of HO-1, NQO1, GCLC and GCLM, and the results demonstrated that Nrf2 knockdown can inhibit the expression of anti-oxidative stress genes (Fig. 9F–I). These results showed that the protective effect of DJ-1 against oxidative stress was weakened under DJ-1 deficiency.

Taken together, these data indicate that DJ-1 can regulate antiapoptotic and anti-oxidative stress mechanisms in RRPs through the Nrf2 signaling pathway.

## Discussion

Along with the increasing number of patients with DR, there is a growing awareness of the importance of retinal protection in early diabetes^19^. Our previous study reported that oxidative stress plays an important role in DR, and increasing the levels and activity of retinal MnSOD and CAT can effectively prevent the progression of DR^20^. Other emerging reports have confirmed that DJ-1 may participate in anti-oxidative stress mechanisms through multiple pathways; for example, DJ-1 may regulate antiapoptotic genes and anti-oxidative stress genes^21,22^. However, the specific anti-oxidative stress mechanism has still not been fully defined. With regards to ocular diseases, studies have shown that DJ-1 plays an antioxidative role in Fuchs corneal endothelial dystrophy and age-related macular degeneration^16,23^, but whether DJ-1 can regulate oxidation in DR has not been studied. Therefore, whether DJ-1 regulates oxidative stress in DR and the specific mechanism are worthy of further investigation.

Our results showed that ROS and apoptosis were increased in RRPs under high-glucose conditions. Apoptosis can be regulated by both antiapoptotic genes and apoptotic genes. Common antiapoptotic proteins include Bcl-2 and Bcl-xl, while common apoptotic proteins include BAX and Bak. It is generally believed that the ratio of Bcl-2 to BAX determines the survival or apoptosis of cells stimulated by apoptotic factors^24^. The ratios of Bcl-2 to BAX in this study were consistent with the trends in apoptosis. The results indicated that oxidative stress was increased and that the activity of antioxidant enzymes, including MnSOD and CAT, was decreased in RRPs stimulated by high glucose; however, the expression of these enzymes increased within the 2-day exposure period, which may have been due to an enzymatic compensatory mechanism in the early stages of high glucose exposure^25^. The activity of antioxidant enzymes is low under normal physiological conditions, but the expression of the enzymes can increase when cells are stimulated by high glucose, which induces oxygen free radical overproduction and ROS accumulation. However, oxidative modification or glycosylation under high-glucose conditions can decrease antioxidant enzyme activity, and studies have shown that high glucose-induced oxidative stress can exceed protein tolerance, causing protein conformational changes to occur^26^. Therefore, the expression of antioxidant enzymes was increased in the early stage, but the activity of these enzymes was decreased. ROS overproduction caused an imbalance between ROS production and clearance, which in turn led to activation of apoptotic pathways.

After DJ-1 overexpression, the expression of the apoptotic protein BAX was decreased, and the expression of the antiapoptotic protein Bcl-2 was increased. When RRPs were exposed to high glucose, oxidative stress increased, the mitochondrial membrane potential was depolarized, mitochondrial membrane transport pores were opened, and mitochondrial membrane permeability increased; thus, cytochrome C, BAX and other apoptosis-inducing factors leaked from the mitochondria into the cytoplasm, activating caspase-dependent and caspase-independent apoptosis^27^. A previous study showed that DJ-1 can protect mitochondrial function and structure by directly binding to two subunits encoding mitochondrial complex I located in the nucleus and mitochondria and by acting as a constitutive protein of mitochondria colocalized with mitochondrial complexes that maintains the activity of mitochondrial complex I^28,29^. Therefore, in the early stage of DR, DJ-1 may be transported to the mitochondria and inhibit mitochondrial membrane potential depolarization, thereby stabilizing membrane permeability, preventing the release of apoptotic and proapoptotic factors and playing a protective role in RRPs.

After DJ-1 overexpression, the expression levels of phase II metabolic enzymes, such as HO-1, NQO1, GCLC and GCLM, were increased. Those enzymes play vital roles in detoxification processes in the body^30^. Studies have shown that HO-1 can decompose free hemoglobin and is related to stress damage in the body, preventing excessive accumulation of free heme and producing carbon monoxide (CO), bilirubin and H-ferritin. These products exert protective effects against oxidative stress. There have been different reports on the expression changes in HO-1 and NQO1 under high-glucose conditions. Some studies have suggested that HO-1 can be induced by oxidative stress in DR; however, the expression levels of HO-1 are different in different stages of diabetes, and long-term persistent hyperglycemia can decrease HO-1 expression^19,31^. These differences may be related to the different detection times of HO-1 and NQO1 protein expression and the different glucose concentrations and detection times for high-glucose cell cultures in these studies; however, we found that the trends in HO-1 and NQO1 expression were consistent with the changes in Nrf2 expression. Our study showed that the expression of HO-1, NQO1, GCLC and GCLM was increased in RRPs exposed to 30 mmol/L glucose within 2 days, and the expression of Nrf2 was also increased. This effect may have been due to excessive ROS-induced expression of Nrf2 and its downstream phase II metabolism enzymes. However, long-term exposure to high glucose depleted Nrf2, HO-1, NQO1, GCLC and GCLM protein levels and weakened anti-oxidative stress mechanisms, leading to apoptosis of RRPs.

The Nrf2 pathway has been studied in a variety of diseases, and it has also become an important target for research on diabetic diseases and related complications. Experimental studies have confirmed that knockout of the Nrf2 gene can aggravate the development of type 1 and type 2 diabetes. Nrf2-knockout mice exhibit reduced oxidative stress resistance, decreased ROS clearance, increased inflammatory factor levels, and increased apoptosis. Therefore, we believe that Nrf2 activators can protect against diabetes^32,33^. In diabetic cardiovascular complications, chronic inflammation is closely related to oxidative stress factors induced by hyperglycemia. Activation of the Nrf2-ARE signaling pathway can prevent high glucose-induced endothelial cell biochemical dysfunction; therefore, Nrf2 activators may be of value in the treatment of diabetic cardiovascular complications^34,35^. Studies have shown that the binding of retinal Nrf2 to Keap1 was increased in DR rats, Nrf2 DNA-binding activity and Nrf2 binding to the GCLC promoter region was decreased. When retinal vascular endothelial cells are exposed to high glucose, they can be damaged because of decreases in retinal antioxidative activity. However, this damage can be prevented by the Nrf2 inducer t-butylhydroquinone (tBHQ) and Keap1 siRNA^36^. Therefore, based on the above findings, inhibition of Nrf2 reduction or activation of Nrf2 may be targets for the treatment of DR. DJ-1 overexpression enhanced the expression of Nrf2 and its downstream phase II metabolic enzymes, including HO-1, NQO1, GCLC and GCLM. In contrast, when Nrf2 was inhibited, even if DJ-1 was overexpressed, the expression of phase II metabolic enzymes was not significantly increased, and RRPs apoptosis was not significantly reduced. We hypothesize that DJ-1 may activate the dissociation of Nrf2 and Keap1 and that the dissociated Nrf2 enters the nucleus and binds to AREs, initiating transcription of ARE-regulated genes and thereby increasing the activity of downstream phase II metabolic enzymes to reduce oxidative stress and prevent apoptosis in RRPs.

In summary, this study indicated that DJ-1 can play an antiapoptotic and anti-oxidative stress role in RRPs exposed to high glucose and confirmed that DJ-1 can regulate apoptosis and oxidative stress in RRPs through the Nrf2 signaling pathway to ultimately reduce high glucose-induced apoptosis. Our current study results suggest that DJ-1 may be an intervention target for early DR that plays a protective role in the retina. Enhancing DJ-1 may reduce retinal capillary pericyte apoptosis, thus delaying or preventing the development of early DR.

## Methods

### Primary culture of rat retinal pericytes (RRPs)

All experiments were approved and conducted in accordance with the Institutional Animal Care and Use Committee at the Second Xiangya Hospital, Central South University. Studies were conducted in accordance with the ARVO Statement Principles for the Use of Animals in Ophthalmic and Vision Research. The Sprague-Dawley (SD) rats were killed with an intraperitoneal injection of 20% chloral hydrate and the eyes were enucleated. After incision of the eyes along the ora serratae, the cornea, lens, and vitreous body were removed. The entire retina was gently peeled away and place it in D-Hanks solution to rinse. After cutting the retina fully, the homogenate was centrifuged and the pellet was resuspended in DMEM containing 0.2% collagenase I. After digestion (50 min at 37 °C), fragments of microvessels were collected on a sieve (100 μm mesh) and seeded on fibronectin-coated dishes (100 μg per 6 cm diameter dish). RRPs cultures were nearly 100% pure as identified by immunocytochemical staining.The eluate was collected using a 50 ml graduated centrifuge tube, centrifuged at 1000 rpm for 5 min at 4 °C, and the supernatant was aspirated. The sediment was resuspended in low-glucose (containing 1 g/L D-Glucose, Gibco) dulbecco’s modified eagle medium (DMEM) medium containing 20% fatal bovine serum (FBS). Cells were cultured in low-glucose DMEM and seeded in collagen type IV-coated plates and flasks. When cells reached 90% confluence, endothelial cells were separated from pericytes by short trypsinization (0.05% trypsine ethylene diamine tetraacetic acid (EDTA); Invitrogen). After addition of trypsine EDTA, the majority of endothelial cells were detached and the remaining pericytes were washed twice in PBS and supplemented with low-glucose DMEM. Cells were cultured until confluence^37,38^.

### Cell transfection

After an overnight incubation, the attached RRPs were washed twice and incubated with serum-free DMEM containing the indicated concentrations of glucose for specific times. The cells were incubated in 10-cm culture dishes for proliferation, and the medium was replaced every 2 days. Subsequently, enough cells were seeded in specific culture dishes for further experiments. According to the different purposes of the experiments, the experimental cells were divided into different groups: a normal control group (NC group, cultured with 5.5 mmol/L glucose), a high-glucose group (HG group, cultured with 30 mmol/L glucose), a hypertonic group (HP group, cultured with 5.5 mmol/L glucose and 24.5 mmol/L mannitol), a DJ-1-overexpressing group treated with high glucose (DJ-1 overexpression group), an empty vector-transfected group treated with high glucose (empty vector), an Nrf2-silenced group treated with high glucose (HG + Nrf2 siRNA), and a DJ-1-overexpressing and Nrf2-silenced group treated with high glucose (HG + DJ-1 + Nrf2 siRNA). All RRPs were cultured for 2 days in the different media.

### TUNEL staining

TUNEL staining was carried out using a fluorescein *In Situ* Cell Death Detection Kit (Roche Diagnostics, Mannheim, Germany) according to the manufacturer’s protocol. Briefly, cells were washed with phosphate buffer saline (PBS) and fixed in freshly prepared 4% paraformaldehyde in PBS. Fixation was followed by a 2-min incubation with ice-cold permeabilization solution (0.1% Triton X-100, 0.1% sodium citrate). The cells were then rinsed twice with PBS and incubated for 1 hour at 37 °C with the TUNEL reaction mixture. After incubation, the cells were rinsed three times with PBS, mounted on microscope slides, and photographed using a fluorescence microscope (Nikon Instruments Inc., Melville, NY, USA). The percentage of apoptotic cells/total cells (100 cells) in five fields was determined in three independent experiments.

### Quantification of ROS

Cellular ROS production was evaluated by measuring the oxidative conversion of cell-permeable dichlorodihydrofluorescein diacetate (DCFH-DA; Beyotime Institute of Biotechnology) to green fluorescent 2’,7’-dichlorofluorescein (DCF;). DCFH-DA was dissolved in dimethyl sulfoxide to a final concentration of 5 μM. RRPs (1 × 10^6^ cells/ml) were incubated with 2 mM DCFH-DA for 20 min under standard conditions, washed three times in PBS and resuspended. The RRPs were assessed with a flow cytometer (FACScan; BD Biosciences) equipped with FACSComp software (Quadra 650) at excitation/emission wavelengths of 488/525 nm.

### Western blot analysis

Western blot analysis was performed with standard methods. Briefly, cells were collected in sodium dodecyl sulfate (SDS) lysis buffer with protease inhibitor cocktail (Sigma; 1:100) and phosphatase inhibitors (Roche; 1:100). The cell samples were crushed with an ultrasonic pulverizer, and the protein concentrations were quantified using a bicinchoninic acid (BCA) protein quantitative kit (Multisciences Biotech Co. Ltd, Hangzhou, China). Then, 35~40 μg of total proteins were separated by 12% SDS-polyacrylamide gel electrophoresis (SDS-PAGE) and electrotransferred to polyvinylidene fluoride (PVDF) membranes (Merck Millipore, MA, USA). After blocking with 5% skim milk for 1 hour at room temperature, the membranes were incubated at 4 °C overnight with the following specific antibodies (1:1000): DJ-1 (Cell Signaling Technology, #5933), SOD2 (Abcam, ab13533), CAT (Cell Signaling Technology, #14097), Bcl-2 (Abcam, ab59348), BAX (Abcam, ab32503), HO-1 (Abcam, ab68477), NQO1 (Abcam, ab28947), GCLC (Abcam, ab53179), GCLM (Abcam, ab124827), Keap1 (Abcam, ab666620), Nrf2 (Abcam, ab137550) and GAPDH (SANTA, SC-365062). After washing with 0.1% PBS with Triton X-100 (PBST) thrice, the membranes were incubated at room temperature for 1 hour with horseradish peroxidase (HRP)-conjugated goat anti-rabbit (CWbiotech, China; CW0103S) or anti-mouse (ZSGB-Bio, China; ZB-5305) IgG (1:10000). Then, the blots were washed with 0.1% PBST thrice and incubated with western blotting luminescent solution (Millipore, MA, USA). The densities of the bands were detected with a Gel Doc 1000 imaging analysis system (Bio-Rad, CA, USA) and analyzed with ImageJ software (National Institutes of Health, http://rsb.info.nih.gov/ij/). The integrated optical density (IOD) of each band was calculated and normalized to that of reduced glyceraldehyde-phosphate dehydrogenase (GAPDH)(to yield the relative IOD, RIOD).

### Design and screening of Nrf2 siRNAs

RNA interference of Nrf2 was performed using 21-bp siRNA duplexes purchased from Gima Corporation (Shanghai, China). The siRNAs for Nrf2 were designed as follows:

#1, GGGUAAGUCGAGAAGUGUUTT (sense) and

AACACUUCUCGACUUACCCTT (antisense);

#2, CCGGAGAAUUCCUCCCAAUTT (sense) and

AUUGGGAGGAAUUCUCCGGTT (antisense); and

#3, UGCCCACAUUCCCAAACAATT (sense) and

UUGUUUGGGAAUGUGGGCATT (antisense).

The negative control siRNA sequences were UUCUCCGAACGUGUCACGUTT (sense) and ACGUGACACGUUCGGAGAATT (antisense).

The cells were transfected with 10 nM siRNA duplexes using Lipofectamine RNAiMAX (Invitrogen, Carlsbad, CA, USA) according to the manufacturer’s instructions. Then, we used western blot analysis to detect the efficiency of Nrf2 silencing.

### Statistical analysis

The results are expressed as the mean ± SEM and were analyzed with SPSS 22.0 software (SPSS Inc., Chicago, IL, USA). Each experiment was repeated three times. The Shapiro-Wilk test was used to test normality. Differences among groups were analyzed using one-way ANOVA followed by Tamhane’s test and the least significant difference (LSD) method. The Kruskal-Wallis test was used when data did not conform to a normal distribution. P < 0.05 was considered to indicate statistical significance.

## Data Availability

The datasets generated during and/or analyzed during the current study are available from the corresponding author on reasonable request.
